# Tandem Mass Tags Quantitative Proteomics Reveal the Mechanism by Which Paeoniflorin Regulates the PI3K/AKT and BDNF/CREB Signaling Pathways to Inhibit Parkinson’s Disease

**DOI:** 10.3390/ijms26136498

**Published:** 2025-07-06

**Authors:** Zhen Feng, Chang Jin, Yue Zhang, Huiming Xue, Yongxing Ai, Jing Wang, Meizhu Zheng, Dongfang Shi

**Affiliations:** 1 College of Life Sciences, Changchun Normal University, Changchun 130032, China; fengzhen0915@163.com (Z.F.); j1504408478@163.com (C.J.); 15942907284@163.com (Y.Z.); 13844637912@163.com (H.X.); 2 College of Animal Science, Jilin University, Changchun 130062, China; aiyx@jlu.edu.cn; 3 Technical Innovation Laboratory for Research and Development of Economic Plants and Edible and Medicinal Fungi in Cold Regions of Jilin Province, Changchun Normal University, Changchun 130032, China; ccsfxy683@126.com; 4 Central Laboratory, Changchun Normal University, Changchun 130032, China

**Keywords:** proteomics, paeoniflorin, Parkinson’s disease, BDNF/CREB signaling pathway, PI3K/AKT signaling pathway

## Abstract

Paeoniflorin (PF), a monomeric compound extracted from the dry roots of *Paeonia lactiflora*, has been widely used in the treatment of nervous system diseases, marking it as a critical formula in Parkinson’s disease (PD). However, the action of PF against PD and its molecular mechanism are still unclear. In this study, tandem mass tags quantitative proteomics was performed to systematically clarify the underlying mechanism of action of PF against PD and to confirm it using in vivo and in vitro studies. The results showed that PF notably enhanced the viability of PC12 cells and mitigated MPP^+^-induced mitochondrial dysfunction, oxidative stress, and apoptosis. Tandem mass tag-based quantitative proteome analysis revealed the identification of 6405 proteins, of which 92 were downregulated and 190 were upregulated. Among them, the levels of PI3K, AKT, CREB, and BDNF in the MPP^+^-induced PC12 cell and MPTP mice were considerably lower than in the control group, indicating the role of the BDNF/CREB pathway in the pathogenesis of neuroprotection. The related DEP (PI3K, AKT, CREB, and BDNF) expression levels were verified by Western blot. PF effectively restored the altered expression of the four DEPs induced by MPP^+^ and MPTP. Summarily, PF exerted remarkable neuroprotective effects in MPP^+^-induced PC12 cell and MPTP-induced mice. Further, our research may provide proteomics insights that contribute to the further exploration of PF as a potential treatment for PD.

## 1. Introduction

As the second most common neurodegenerative disorder after Alzheimer’s disease, Parkinson’s disease (PD) prevalence is projected to increase by 50% by 2030, paralleling global trends of population aging and modern lifestyle stressors [1,2,3]. The progression of PD increasingly impairs patient quality of life, imposing significant burdens on individuals, their families, and society at large [4]. Consequently, there is a pressing need to identify efficacious treatments for Parkinson’s disease and to elucidate its underlying mechanisms.

Investigations have established a potential link between the mechanisms of synaptic plasticity and the emergence of neurological conditions, identifying a correlation between the deficiencies in synaptic plasticity and a shortfall in essential nutrients, including BDNF (brain-derived neurotrophic factor) [5,6]. The scarcity of neurotrophic support might compromise the cognitive capabilities and adaptability of the brain, which could be a key contributing factor to the development of diseases affecting the central nervous system [7]. It has a significant role in the healthy development of neurons, including their survival, by influencing neuronal survival and synaptic plasticity through the interaction with certain receptors [8]. Phosphorylation processes following the pairing of BDNF with its receptor, TrkB (tyrosine kinase receptor B), activate PI3K/Akt pathway, which allows signal transmission to the nucleus. This event induces CREB, a nuclear transcription factor and activator of the proliferation and differentiation of neurons [9]. Stimulation of the signaling cascade involving CREB (cAMP-response element binding protein), BDNF, and TrkB enhances the prospects for treatment of neurological conditions through pharmacological methods or alternative approaches. Moreover, the BDNF-TrkB signaling pathway is essential for the survival of dopaminergic neurons in the substantia nigra striatum, and its production is tightly regulated by neural activity. Disruption of this neurotrophic pathway contributes significantly to PD development by compromising the survival and maintenance of dopaminergic neurons. Reduced BDNF levels in the substantia nigra are believed to be the main cause of the degeneration of dopaminergic neurons in PD patients because of insufficient trophic support [10]. Moreover, BDNF plays a crucial role in protecting dopaminergic neurons in the substantia nigra by counteracting MPTP-induced disruption of tyrosine hydroxylase (TH) biosynthesis, thereby preserving catecholamine production in this brain region [11,12].

*Paeonia lactiflora* Pall., a plant from the family Ranunculaceae, has been extensively utilized in traditional Chinese medicine (TCM), where its dried root (*Radix Paeoniae* Alba) serves as an important herbal drug with multiple pharmacological activities. For centuries, it has been widely used in China to alleviate pain, reduce inflammation, and treat immune-related diseases [13,14]. A monomeric entity taken from the *Radix Paeoniae* Alba, PF falls into the category of monoterpenoids. PF is a vital component in therapies for rheumatic and autoimmune diseases, hepatitis, and liver cirrhosis and has attracted the attention of the healthcare industry [14,15]. Pharmacological research has proven PF to relieve inflammatory pain, suppress oxidative stress, modulate the immune system, promote autophagy, and inhibit apoptosis [16,17]. On account of its wide range of biological effects, PF is extensively used in the treatment of nervous system disorders such as PD, cerebral ischemia, hypoxia, and Alzheimer’s disease [18]. In this study, Madopar (a combination of levodopa and benserazide) was selected as the positive control drug due to its well-established efficacy in treating Parkinson’s disease (PD) by replenishing dopamine levels in the brain [19]. Other neuroprotective agents, such as rasagiline, an irreversible selective monoamine oxidase-B inhibitor that has demonstrated potential in slowing disease progression [20], and coenzyme Q10, an antioxidant that has shown promise in early clinical trials [21], were not chosen for this study. The primary reason for not selecting these agents is that our focus was on comparing the neuroprotective effects of paeoniflorin (PF) with a more commonly used and clinically relevant treatment (Madopar) in PD models. Additionally, the neuroprotective mechanisms of rasagiline and coenzyme Q10 are relatively well-understood, whereas the comprehensive proteomics analysis in this study aims to elucidate the novel molecular pathways regulated by PF.

**Figure 1:**
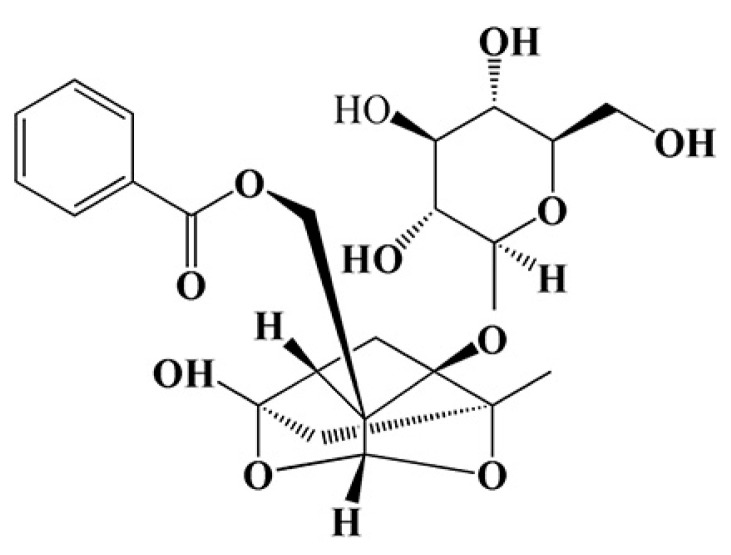
Structural representation of paeoniflorin.

Using TMT-based quantitative proteomics, we systematically identified molecular alterations induced by PF in Parkinson’s disease (PD) models. These findings were rigorously validated through comprehensive in vitro (MPP+-induced PC12 cells) and in vivo (MPTP-treated mice) experiments, demonstrating PF’s consistent neuroprotective efficacy across experimental paradigms. Moreover, our study systematically elucidates the molecular targets and signaling pathways modulated by paeoniflorin (PF), providing mechanistic insights into its neuroprotective actions. These findings significantly advance both the theoretical framework and translational potential of PF-based therapeutics for Parkinson’s disease (PD). By delineating PF’s polypharmacology, our work establishes a foundation for developing targeted therapeutic strategies to ameliorate PD progression and improve clinical outcomes (Figure 1).

## 2. Results

### 2.1. Protein Omics Analysis Results

#### 2.1.1. TMT Analysis of the Differentially Expressed Proteins

In total, 6405 proteins were identified, and 5525 had quantitative data available. FDR correction was applied to the *p*-values of all statistical tests. Employing a criterion of a 1.5-fold change and a *p*-value less than 0.05, 282 proteins showing differential expression were identified in the comparison between the Model and Cell groups; within these, 190 proteins were found to be increased in expression, and 92 were decreased. Furthermore, in the comparison between the Drug and Model groups, 613 proteins were identified as differentially expressed, with 256 exhibiting increased expression and 357 showing decreased expression.

**Figure 2:**
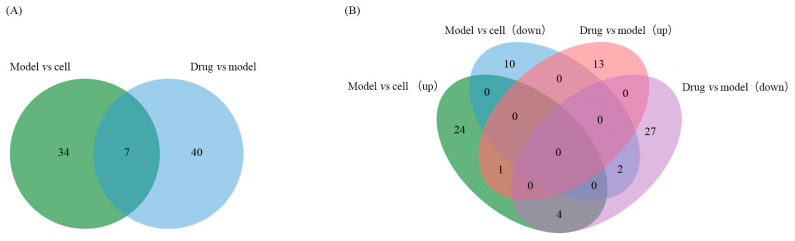
Statistical information of differentially expressed proteins. (**A**) Total amount of differential proteins between the two groups. (**B**) Upregulated differential proteins between the two groups.

Among the quantified proteins, in order to reveal the molecular mechanism of paeoniflorin against Parkinson’s, this experiment was conducted to compare the differential proteins in different groups two by two, which were divided into the Model and Cell comparison group and the Drug and Model comparison group, and screened the proteins with significant differential expression in the two groups as shown in Figure 2A. Proteomics analysis showed that in the Model–Cell comparison group, 41 differentially expressed proteins were identified (*P*_FDR_ < 0.05), of which 29 proteins showed upregulation in expression and 12 proteins showed downregulation; in the Drug–Model comparison group, 47 differentially expressed proteins were identified (*P*_FDR_ < 0.05), of which 14 proteins were upregulated and 33 proteins with downregulated expression (Figure 2B).

By comparing the differential proteins, a total of 77 differential proteins (except unknown proteins) were found to be coexisting in the Model/Cell versus Drug/Model comparison group, of which, 29 differential proteins had opposite expression in the two groups, and the rest of the differential proteins showed consistent changes in expression in the two groups, and the top ten differential proteins with opposite expression have been listed (refer to Table 1).

#### 2.1.2. Gene Ontology Functional Enrichment Analyses of Differentially Significant Expressed Proteins

Gene ontology (GO) is a conventional gene function classification system yielding a list of dynamically updated, standardized functionality terms that enable the functional interpretation of DSEPs. Statistics for differential protein analysis were performed separately for biological processes (BPs), cellular components (CCs), and molecular functions (MFs), and the screening condition was set at *p* < 0.05.

**Figure 3:**
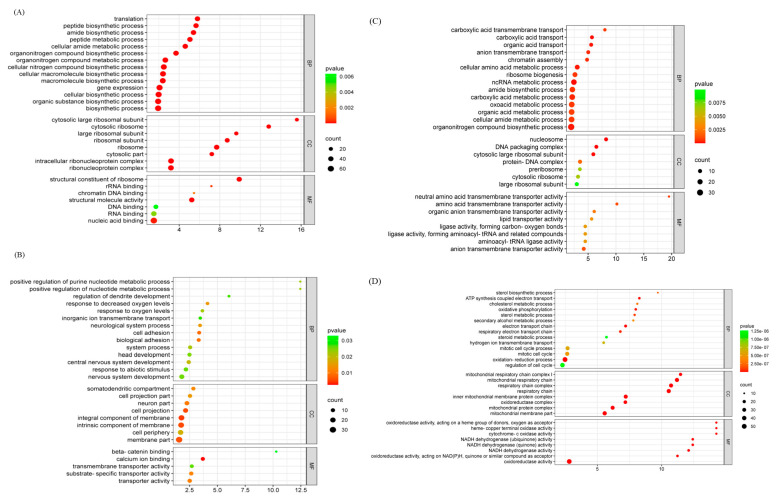
Bubble charts for GO analysis of network pharmacological analyses. (**A**) Up protein pathway enrichment (Model/Cell). (**B**) Down protein pathway enrichment (Model/Cell). (**C**) Up protein pathway enrichment (Model/Drug). (**D**) Up protein pathway enrichment (Model/Cell).

The results demonstrated that all differentially expressed proteins identified in the Model vs. Cell comparison group were predominantly associated with: Biological processes: cellular processes (19%), metabolic processes (14%), and single-organism processes (13%); Molecular functions: binding (54%) and catalytic activity (21%); Other categories: including cellular components and additional functional classifications. All differential protein expressions in the Drug/Model comparison group were mainly involved in biological processes such as cellular processes (18%), single biological processes (15%), and molecular functions such as cellular (50%), organelle (26%), and cell membrane (14%) functions. This analysis led to the identification of 32 statistically significant GO terms, which included eight related to CCs, fourteen to BPs, and ten to MFs, as showcased in Figure 3.

**Figure 4:**
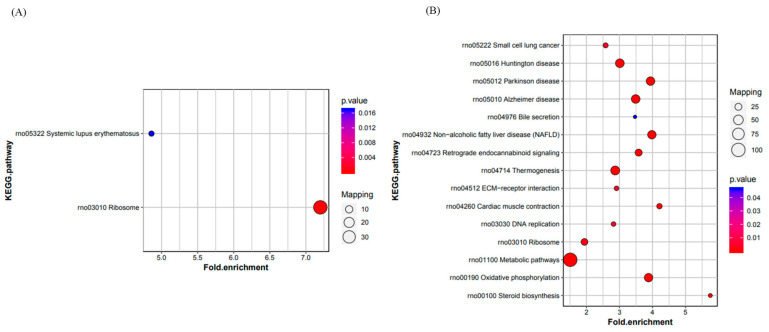
GO enrichment of differential proteins: (**A**) Model/Cell group; (**B**) Drug/Model group.

The entries in the GO annotations, other than the first three major categories, were analyzed for enrichment. The results of the analysis were made into GO enrichment bubble plots by fold enrichment from high to low and top to bottom, with *p* < 0.05 (Figure 4).

The results showed that the differential proteins in the Model/Cell group were involved in six molecular functions, eight cellular components, and fourteen biological processes. The molecular functions include the structural constituent of ribosome, translation regulator activity, rRNA binding, chromatin DNA binding, structural molecule activity, and chromatin binding. Cellular constituents include the cytosolic large ribosomal subunit, nucleosome, cytoplasmic large ribosomal subunit, and cytoplasmic large ribosomal subunit. Biological processes include chromatin assembly or disassembly, translation, peptide biosynthetic process, amide biosynthetic process, etc. (Figure 4A).

The differential proteins in the Drug/Model group involve 8 molecular functions, 8 cellular components, and 14 biological processes. The molecular functions included cytochrome-c oxidase activity, NADH dehydrogenase activity, and oxidoreductase activity, etc. The cellular components included NADH dehydrogenase Cellular components include NADH dehydrogenase complex, mitochondrial respiratory chain and inner mitochondrial membrane protein complex, etc. Biological processes include cholesterol biosynthetic process, sterol biosynthetic process, oxidative phosphorylation, electron transport chain, chromatin assembly (chromosome assembly), and mitochondrial respiratory chain, and inner mitochondrial membrane protein complex, transport chain, chromatin assembly, oxidation-reduction process, and small molecule metabolic process (Figure 4B).

#### 2.1.3. Kyoto Encyclopedia of Genes and Genomes Pathway Enrichment Analysis

**Figure 5:**
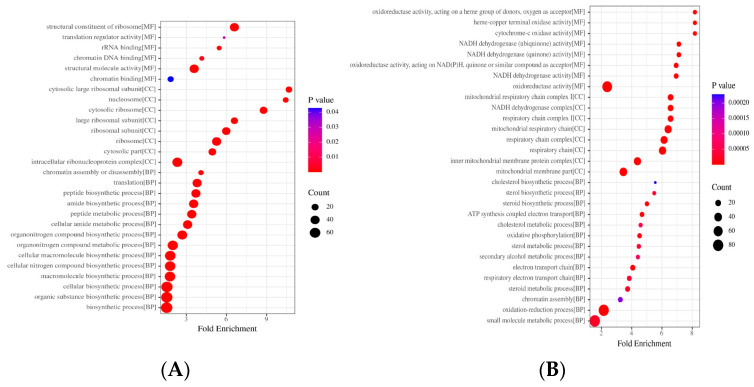
KEGG enrichment of differential proteins: (**A**) Model/Cell group; (**B**) Drug/Model group.

KEGG pathway enrichment analysis showed that 23 pathways were involved in differential protein expression in the Model/Cell group, of which the only two pathways with a *p* value of Fisher’s exact test of less than 0.05 were the ribosomal pathway and the SLE pathway (Figure 5A). A total of 65 pathways were involved in the Drug/Model group, of which 15 pathways with a *p* < 0.05 were steroid biosynthesis, myocardial contraction, non-alcoholic fatty liver disease, Parkinson’s disease, oxidative phosphorylation, ECM–receptor interaction pathways, and so on. Fifteen pathways had *p* < 0.05, including steroid biosynthesis, myocardial contraction, nonalcoholic fatty liver disease, Parkinson’s disease, oxidative phosphorylation, and ECM–receptor interaction pathways (Figure 5B). In addition, the pathways of myocardial contraction, nonalcoholic fatty liver disease, Parkinson’s disease, Alzheimer’s disease, Huntington’s disease, and thermogenesis have common protein involvement with the oxidative phosphorylation pathway.

**Figure 6:**
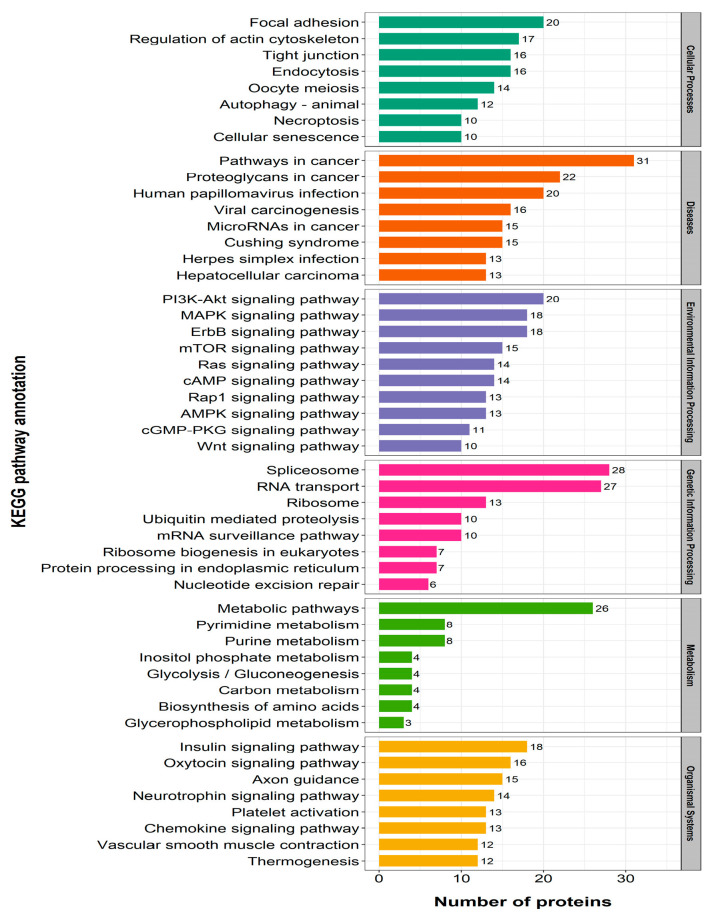
KEGG pathway enrichment.

**Figure 7:**
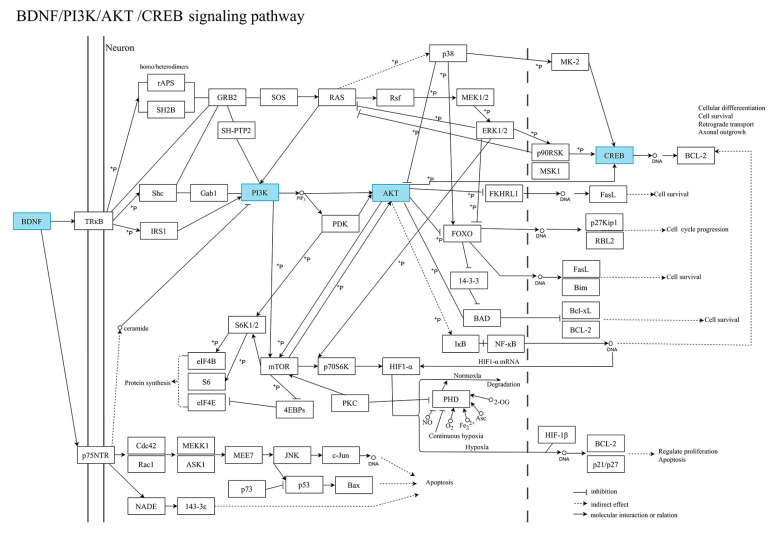
KEGG BDNF/PI3K/AKT/CREB pathway diagram.

Analysis of the KEGG pathway for all differentially expressed proteins revealed significant changes in the PI3K-Akt signaling pathway. In the detailed map of this pathway (Figure 6), BDNF was found to be the most highly associated protein with this pathway. Among the differential proteins identified in this study, BDNF, CREB, PI3K, and Akt are the most important proteins involved in this pathway, detailed in Figure 7.

### 2.2. In Vitro and In Vivo Experiments Results

#### 2.2.1. PF Inhibited MPP^+^-Induced PC12 Cell Death

**Figure 8:**
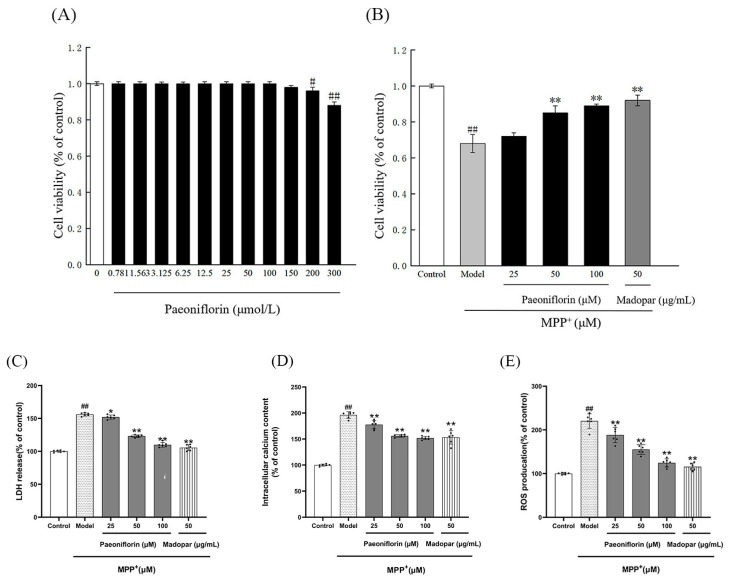

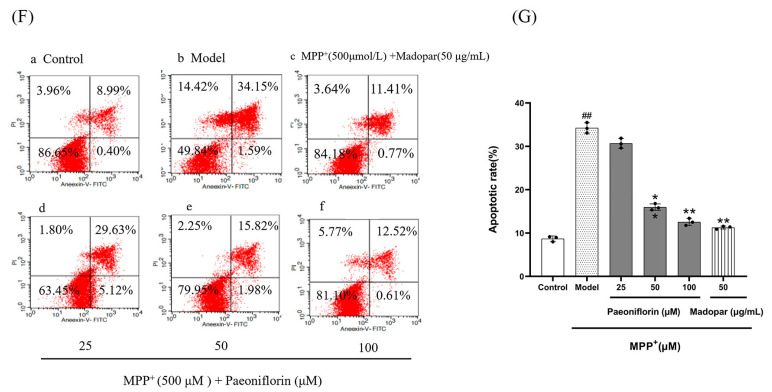
Enhancement of cell viability by PF against MPP^+^ damage. (**A**) Viability of PC12 cells after treatment with varying concentrations of PF (0–300 μM) for 24 h, evaluated through the MTT assay. (**B**) Examination of PF’s protective efficacy (25, 50, 100 μM) on PC12 cells against MPP^+^-induced damage, using the MTT assay. (**C**) Effect of paeoniflorin on MPP^+^-induced LDH release from PC12 cells. (**D**) Effect of paeoniflorin on Ca^2+^ content of PC12 cells induced by MPP^+^. (**E**) Effect of paeoniflorin on ROS content in MPP^+^-induced PC12 cells. (**F**) Flow chart of the effect of paeoniflorin on apoptosis of PC12 cells. a–f represents the apoptosis of each group, and the upper right and lower right quadrants represent early apoptotic cells and late apoptotic and necrotic cells. (**G**) Histogram of the effect of paeoniflorin on apoptosis in MPP^+^ PC12 cells. Data are presented as mean ± SD, with *n* = 5. Not significant (ns) indicates *p* > 0.05, while ^#^ *p* < 0.05, and ^##^ *p* < 0.01 denote significance compared to the control group. * *p* < 0.05 signifies a significant difference from the Model group.** *p* < 0.01 signifies a significant difference from the Model group.

To establish a safe concentration range of PF that does not exert inherent toxicity on PC12 cells (with its chemical structure shown in Figure 8A), doses of PF ranging from 0 to 300 μmol/L were administered to the cells, followed by incubation for 48 h. The MTT assay results demonstrated that concentrations of PF up to 100 μM did not significantly reduce PC12 cell viability (*p* > 0.05). However, a significant decrease in cell viability was observed at higher concentrations of PF (*p* < 0.05), as shown in Figure 8B. Based on these findings, subsequent experiments utilized PF concentrations of 25, 50, and 100 μmol/L.

As illustrated in Figure 7, exposure to MPP^+^ significantly reduced the survival rate of PC12 cells compared to the control group, with the difference being statistically significant (*p* < 0.01). The introduction of PF at concentrations of 50 and 100 μM, as well as Madopar at 50 μM, markedly improved the survival rates. These improvements, in comparison to the MPP^+^-treated group, were statistically significant (*p* < 0.01 for each comparison).

Paeoniflorin pretreatment significantly ameliorated the above impairments. LDH release: paeoniflorin 12.5 μmol/L (*p* < 0.01), 25 μmol/L (*p* < 0.05), and 50 μmol/L (*p* < 0.05) dosage groups significantly reduced LDH leakage suggesting that it protects the integrity of cell membranes. Ca^2+^ levels: paeoniflorin 25 μ mol/L, and 50 μmol/L dose groups significantly alleviated Ca^2+^ overload (both *p* < 0.01), suggesting that it can regulate calcium homeostasis. ROS accumulation: paeoniflorin 12.5–50 μmol/L reduced ROS levels, with 25 μmol/L and 50 μmol/L dose groups having the most significant effects (both *p* < 0.01), confirming its antioxidant effect (Figure 8D–F).

As shown in Figure 8G, the apoptosis rate of PC12 cells in each group was detected by flow cytometry. The experimental data showed that the apoptosis rate in the normal control group was 9.31%, while the apoptosis rate in the MPP^+^-induced Model group was significantly elevated to 35.74% (*p* < 0.01). After pretreatment with paeoniflorin, the apoptosis rate decreased in a dose-dependent manner, with the apoptosis rate of 28.38% in the 12.5 μmol/L group (* *p* < 0.05), decreasing to 18.18% in the 25 μmol/L group (*p* < 0.01), and further decreasing to 12.56% in the 50 μmol/L group (*p* < 0.01).

#### 2.2.2. PF Can Activate PI3K/AKT and BDNF/CREB Signaling Pathways

**Figure 9:**
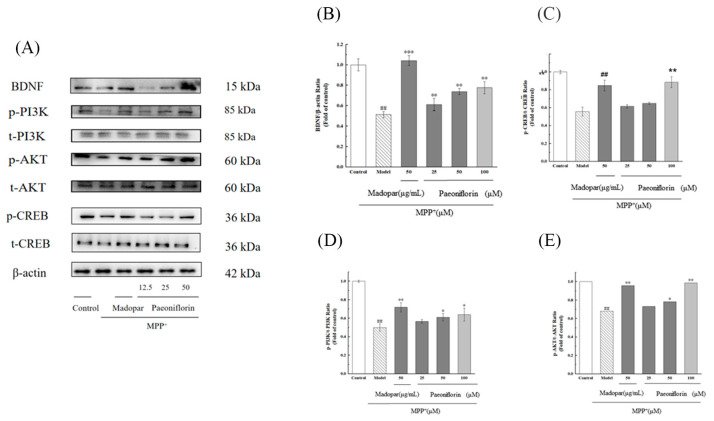
Enhancement of BDNF, phosphorylated PI3K, AKT, and CREB in PC12 cells by PF. (**A**) Analysis of protein expression levels for BDNF, p-AKT, t-AKT, p-PI3K, t-PI3K, p-CREB, t-CREB, and β-actin through Western blot. (**B**) Densitometry for BDNF expression ratios. (**C**) Densitometry for the ratio of phosphorylated to total CREB. (**D**) Densitometry for the ratio of phosphorylated to total PI3K. (**E**) Densitometry for the ratio of phosphorylated to total AKT. Data are reported as mean ± SD, with *n* = 3. Significant differences are denoted as ^##^ *p* < 0.01 compared to the control and as * *p* < 0.05, ** *p* < 0.01 and *** *p* < 0.001 compared to the Model group.

Activation of the PI3K/AKT and BDNF/CREB signaling pathways has been recognized as a key factor in reducing neuronal apoptosis in mouse models of Parkinson’s disease. In this study, the expression levels of proteins including BDNF, phosphorylated and total PI3K (p-PI3K, t-PI3K), phosphorylated and total CREB (p-CREB, t-CREB), and phosphorylated and total AKT (p-AKT, t-AKT) were evaluated. As shown in Figure 9A, the MPP^+^ group exhibited reduced levels of BDNF, p-CREB, p-PI3K, and p-AKT, leading to significant decreases in the ratios of p-CREB/t-CREB, p-PI3K/t-PI3K, and p-AKT/t-AKT (*p* < 0.01 for each). However, administering different doses of PF effectively reversed these reductions, showing a dose-dependent restoration of these proteins, as depicted in Figure 9B–E.

#### 2.2.3. LY294002 Blocks PF’s Neuroprotection via PI3K/AKT and BDNF/CREB Pathway Inhibition

**Figure 10:**
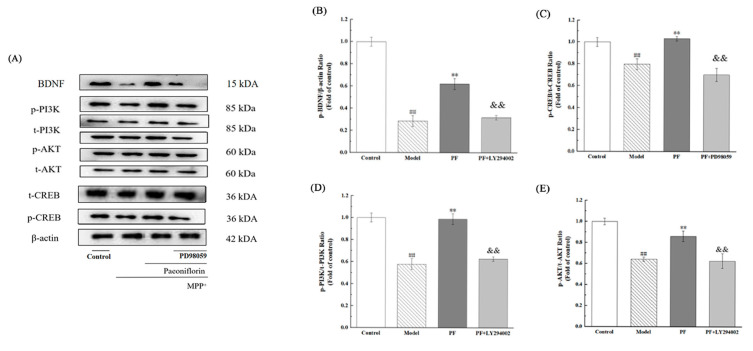
Inhibition of the PI3K/AKT pathway by LY294002 diminishes the neuroprotective effects of PF. (**A**) Evaluation of protein expression for BDNF, p-AKT, t-AKT, p-PI3K, t-PI3K, p-CREB, t-CREB, and β-actin using Western blot analysis. (**B**) Densitometric analysis for the expression ratio of BDNF. (**C**) Densitometric analysis for the phosphorylated-to-total CREB ratio. (**D**) Densitometric analysis for the phosphorylated-to-total PI3K ratio. (**E**) Densitometric analysis for the phosphorylated-to-total AKT ratio. Data are expressed as mean ± SD, with *n* = 3. Statistical significance is indicated as ^##^ *p* < 0.01 compared to the control group and as ** *p* < 0.01 compared to the Model group. Compared with the drug group, ^&&^ *p* < 0.01.

To determine if PF’s neuroprotective effects are mediated through the PI3K/AKT and BDNF/CREB signaling pathways, PI3K activity was inhibited using LY294002. This was followed by measuring the expression levels of BDNF, phosphorylated and total CREB (p-CREB, t-CREB), phosphorylated and total PI3K (p-PI3K, t-PI3K), and phosphorylated and total AKT (p-AKT, t-AKT) via Western blotting (as shown in Figure 10A). Compared to the Model group, the combination treatment group (MPP^+^ + PF + LY294002) showed no significant changes in BDNF expression, or the ratios of p-CREB/t-CREB, p-PI3K/t-PI3K, and p-AKT/t-AKT. However, a significant reduction in the expression of these proteins and ratios was observed in the MPP^+^ + PF + LY294002 group compared to the MPP^+^ + PF group (*p* < 0.01 for all comparisons). These findings suggest that PF activates the PI3K/Akt and BDNF/CREB pathways by increasing BDNF protein expression, though LY294002 attenuated these effects. The results highlight the crucial role of the PI3K/AKT and BDNF/CREB signaling pathways in facilitating PF’s neuroprotective effects (illustrated in Figure 10B–E).

#### 2.2.4. PF Effects upon Behavioral Disadvantages MPTP Induction

**Figure 11:**
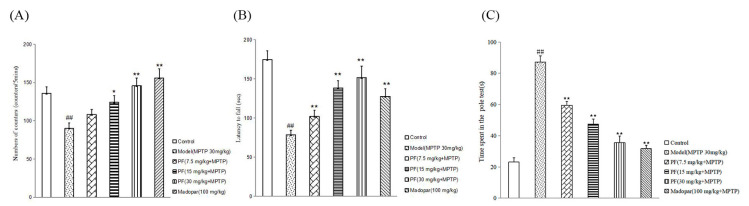
Amelioration of MPTP-induced behavioral impairments by PF. (**A**) Assessment of spontaneous motor activity in mice subjected to MPTP, with and without PF administration. (**B**) Influence of PF on the performance of MPTP-treated mice on the rotarod test. (**C**) Influence of PF on the performance of MPTP-treated mice on the pole test. Statistical significance is denoted as ^##^ *p* < 0.01 relative to the control group and as * *p* < 0.05 and ** *p* < 0.01 compared to the Model group. Measurement scale: 100 µm.

Mice subjected to MPTP treatment displayed a significant decrease in spontaneous motor activities (*p* < 0.01) and a considerable decline in performance on the rotarod test (*p* < 0.01), exhibiting significantly prolonged climbing times (*p* < 0.01), compared to the control group. In contrast, administration of PF at doses of 15 and 30 mg/kg effectively reversed these behavioral deficits, countering the detrimental effects of MPTP-induced toxicity (*p* < 0.01 for each comparison), as depicted in Figure 11.

#### 2.2.5. Effect of PF on MPTP-Induced Reduction in Tyrosine Hydroxylase Immunoreactivity in the Substantia Nigra

**Figure 12:**
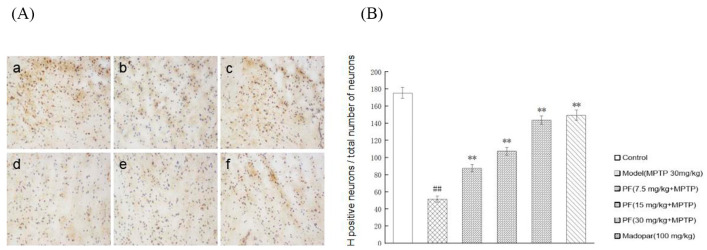
Paeoniflorin’s effect on tyrosine hydroxylase-positive neurons in the substantia nigra of MPTP-induced mouse model. (**A**) Results of immunohistochemical staining for tyrosine hydroxylase in the substantia nigra striata. (**a**–**f**) shows the number of TH-positive neuron cell bodies and fibers of mice in the control group, model group, Medoba group and paeoniflorin 7.5 mg/kg, 15 mg/kg and 30 mg/kg, respectively. (**B**) TH-staining statistics chart. Compared with normal control group, ^##^ *p* < 0.01; compared with Model group, ** *p* < 0.01. (*n* = 10).

Representative microphotographs of TH immunostaining in the substantia nigra are shown in Figure 12. Animals that received vehicle-only treatment following MPTP injection exhibited a significant loss of TH-immunopositive neurons (*p* < 0.05). In contrast, treatment with PF at doses of 7.5, 15, and 30 mg/kg, as well as Madopar treatment, significantly inhibited neuronal loss and increased the number of TH-positive neurons compared to the MPTP group (*p* < 0.01).

#### 2.2.6. In a Mouse Model of Parkinson’s Disease Paeoniflorin Activates the BDNF/CREB Signaling Pathway

**Figure 13:**
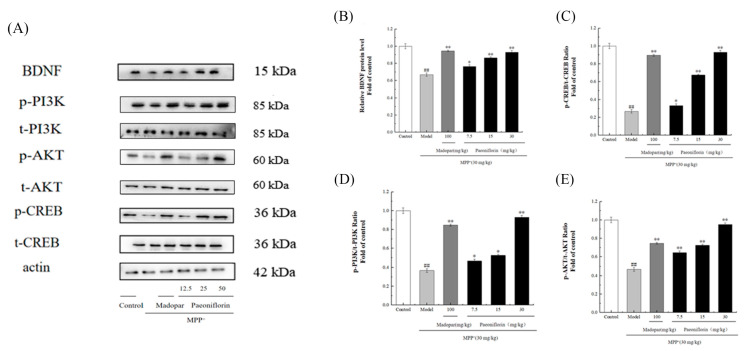
PF enhancement of BDNF, phosphorylated PI3K, AKT, and CREB in a rat model. (**A**) Western blot analysis illustrating expression levels of BDNF, p-AKT, t-AKT, p-PI3K, t-PI3K, p-CREB, t-CREB, and β-actin. (**B**) Densitometric evaluation detailing BDNF expression ratios. (**C**) Densitometric analysis for the ratio of phosphorylated to total CREB. (**D**) Densitometric evaluation focusing on the ratio of phosphorylated to total PI3K. (**E**) Densitometric assessment concerning the ratio of phosphorylated to total AKT. Results are expressed as mean ± SD, for *n* = 3, with ^##^ *p* < 0.01 indicating a significant difference from the control and * *p* < 0.05 and ** *p* < 0.01 signifying statistical significance compared to the Model group.

As depicted in Figure 13, the expression levels of BDNF, phosphorylated PI3K (p-PI3K), phosphorylated AKT (p-AKT), and phosphorylated CREB (p-CREB) in the brain tissues of mice from the Model group were significantly lower compared to the control group. Additionally, the ratios of BDNF to β-actin, p-PI3K to total PI3K (t-PI3K), p-AKT to total AKT (t-AKT), and p-CREB to total CREB (t-CREB) were notably decreased (*p* < 0.01). In contrast, in the group treated with paeoniflorin, these ratios were significantly higher compared to the Model group, mirroring the positive control results obtained with Madopar.

## 3. Discussion

With the demographic shift towards an older population in China, the prevalence of Parkinson’s disease is witnessing a yearly increase. A hallmark of this ailment is its relentless progression, accompanied by the absence of a known cure and a high incidence of disability. These characteristics contribute to significant challenges in the management and treatment of the condition [22,23]. The urgent necessity to identify potential therapeutics for Parkinson’s disease is evident. In the domain of healthcare, traditional Chinese medicine and natural products are distinguished by their gentle nature, minimal toxicity, and the absence of significant adverse effects. These products are notable for their multifaceted mechanisms, which target diverse pathways in the body [24,25,26]. Among these, Radix Paeoniae Alba stands renowned for its pharmacological benefits, including anti-inflammatory properties, liver protection, pain relief, and blood nourishment [27,28]. It is noteworthy that the total glucosides from *Paeonia lactiflora*, a critical extract component, have been recognized for their protective effects on the nervous system and cardiovascular health. Paeoniflorin, as the predominant active constituent, is largely responsible for the neuroprotective actions attributed to *Paeonia lactiflora* [29,30]. However, the intricate compositions and multi-target nature of traditional Chinese medicine complicate the detailed exploration of specific mechanisms at a molecular level. In view of this, our study aims to investigate the neuroprotective potential of PF against brain injury, especially in the context of PD, by analyzing the revealing mechanism of tandem mass labeling quantitative protein omics. This comprehensive approach included analyses such as PPI networks, GO analysis, KEGG pathway enrichment, and molecular docking. Following these analyses, we pursued experimental validations using in vivo and in vitro models that simulate PD conditions. Our integrative investigation sheds light on the targeted genes and pathways by PF, enriching the knowledge base for developing advanced treatments to facilitate recovery following PD.

In our research, we employed the PC12 cell line, distinguished for its neuroendocrine characteristics, to create a model of Parkinson’s disease by administering 500 μmol/L of MPP^+^. This configuration enabled an initial evaluation of the effectiveness of PF in addressing Parkinson’s disease by assessing various cellular health indicators. The metrics encompassed cell survival rates, lactate dehydrogenase (LDH) release, reactive oxygen species (ROS) levels, intracellular calcium concentrations, and apoptosis rates. LDH, an oxidoreductase known for its stability within the cellular environment, is minimally released under normal conditions but sees a significant increase upon cell membrane damage or necrosis, indicating cellular distress [31]. Our data demonstrated a reduction in LDH levels in the culture medium of the PF-treated groups compared to the Model group, suggesting that PF attenuates the membrane damage inflicted by MPP^+^ [32]. The primary site for ROS generation is mitochondria. Excessive ROS accumulation can lead to mitochondrial swelling, collapse of the mitochondrial membrane potential, calcium overload, and, ultimately, cell apoptosis [33]. For ROS detection, we utilized the fluorescent probe DCFH-DA. The interplay between mitochondrial dysfunction and oxidative stress disrupts calcium homeostasis. An excessive influx of calcium, especially harmful to the dopaminergic neurons, increases cell membrane permeability and intracellular ROS levels, initiating apoptosis [34]. Our experimental outcomes indicated that MPP^+^-induced damage elevates intracellular calcium and ROS levels, thereby heightening apoptosis. Contrastingly, cells treated with PF exhibited significantly lower levels of intracellular ROS and calcium, alongside reduced apoptosis rates, compared to the Model group.

This investigation highlighted PF’s potential to mitigate cellular damage induced by MPP^+^, enhancing cell viability and reducing apoptosis, thereby providing a protective effect. Further experimental findings corroborated PF’s role in augmenting the survival rates of PC12 cells subjected to MPP^+^ injury.

Proteomics, the comparative study focusing on protein expression, delves into disease pathogenesis or the mechanisms by which drugs mediate therapeutic effects. This research encompasses the analysis of protein expression in diseased states and post medication, as well as protein interactions [35]. A hallmark of proteomics is the identification and screening of proteins that are differentially expressed across study groups, followed by an in-depth information analysis [36]. In this investigation, we employed tandem mass tag (TMT) labeling along with high-resolution liquid chromatography–mass spectrometry for a quantitative proteomics study. Through comprehensive protein dataset analysis and bioinformatics, we observed significant protein expression changes in the MPP^+^-induced PC12 cell model pre- and post-PF treatment. Notably, the expression levels of proteins such as AKT, MAPK14, and PIK3C2 in the PC12 cells exhibited considerable variation between the Model group/Cell group and the PF group/Model group. The neuroprotective effects of PF against Parkinson’s disease could be associated with the modulation of the brain-derived neurotrophic factor (BDNF) and the activation of the downstream AKT/PI3K signaling pathway.

Brain-derived neurotrophic factor (BDNF) is pivotal in orchestrating the development, maturation, and synaptic networking of neurons and glial cells, acting as a key regulatory protein [37,38]. It interacts with its receptor, Tropomyosin-Related Kinase B (TRKB), which is endowed with inherent tyrosine kinase capabilities. Research underscores the BDNF-TrkB axis as critical for enhancing synaptic growth, plasticity, and neuron survival in the central nervous system, effectively counteracting apoptotic processes. TRKB activation leads to activation of Phosphatidylinositol Kinase (PI3K), and this further activates the downstream cascade of AKT/PI3K signaling. Phosphorylation of cAMP response element binding protein (CREB) emerges as a key process of gene transcription regulation [39]. This phosphorylation process by CREB is essential in controlling the signal transduction pathways that affect the upstream gene expression of BDNF. This modulatory impact includes neuronal plasticity and neurotransmitter synthesis and plays a role in determining synaptic plasticity covering processes like neuro cells proliferation and differentiation [40,41].

The PI3K/AKT pathway, a pivotal component of the effects resulting from the interaction between BDNF/TrkB, functions as the primary anti-apoptotic mechanism. This pathway plays a critical role in neuronal survival, synaptic plasticity, and the mimetic effects of antidepressants [42]. PI3K initiation represents a critical survival line to cells in order for them to escape apoptotic damage [43]. This action activates AKT phosphorylation upregulating defense against apoptosis and maintenance of brain function [44].AKT-mediated phosphorylation inactivates inhibitors of apoptosis, thereby promoting cell survival [45,46]. This result indicates that the PI3K/Akt pathway may be one of the main ways through which PF reduces the toxic influence of MPTP/MPP^+^ on neural cells. The next topic to research will be the BDNF/CREB and PI3K/AKT modulation effects of PF’s protection against Parkinson’s disease, and, in particular, the determination of the expression levels of proteins such as AKT, P-PI3K, and AKT2.

Following this, the study continued in vitro modeling to assess the potential of paeoniflorin (PF) to antagonize Parkinson’s disease at the protein level. The experimental data obtained suggested that after MPP^+^ stimuli-induced damage, the phosphorylation kinetics of the BDNF, PI3K, AKT, and CREB proteins were suppressed. Nevertheless, pre-treatment with PF significantly increased the phosphorylation of these proteins, highlighting its possible neuroprotective effect. In order to investigate this mechanism, the pathways of PI3K/AKT and BDNF/CREB signaling were suppressed through the use of LY294002, a specific inhibitor. The results demonstrated a reduction in the neuroprotective effect of PF, highlighting the importance of BDNF/CREB and PI3K/AKT pathways in the neuroprotection provided by PF. Although our findings highlight the central role of PI3K/AKT in PF neuroprotection, the potential crosstalk between PI3K/AKT and BDNF/CREB warrants further exploration. Future studies using dual-pathway inhibition (e.g., PI3K + BDNF/TrkB blockade) will elucidate synergistic or compensatory mechanisms, especially in vivo. The technical challenges associated with combined inhibitor toxicity will be carefully optimized to ensure mechanistic clarity

In this study, we employed quantitative proteomics to identify differentially expressed proteins (DEPs) and subsequently focused on the PI3K/AKT signaling pathway based on its prominent representation in our dataset and significant enrichment in KEGG pathway analysis. Our proteomic analysis also revealed alterations in other proteins, including α-crystallin B, BAD, and MAP4, which may represent either secondary adaptive responses or novel mechanistic targets. These proteins, implicated in apoptosis regulation and cytoskeletal dynamics, warrant further investigation to elucidate their potential roles in paeoniflorin (PF)-mediated neuroprotection. Additionally, KEGG enrichment analysis identified modulated pathways such as MAPK14 signaling, oxidative phosphorylation, and chromatin remodeling, which may contribute to PF’s pleiotropic effects. However, pharmacological inhibition experiments using LY294002 suggest that these pathways likely play ancillary roles compared to the central PI3K/AKT axis. Future studies integrating multi-omics approaches or combinatorial pathway inhibition will be essential to fully delineate PF’s multi-target network and hierarchical mechanism of action.

The present study employed an in vivo mouse model of MPTP-induced Parkinson’s disease (PD), as previously described in the literature [47], to elucidate the protective activity of PF. Following MPTP administration, this compound is able to cross the blood–brain barrier and selectively affect dopaminergic neurons in the substantia nigra and striatum, leading to the production of MPP^+^ by glial cells [48,49]. MPP^+^ particularly interferes with mitochondrial respiratory processes and induces the generation of oxygen radicals that destroy DA neurons and reduce their number. This is followed by the occurrence of signs that revolve around tremors, reduced activity, decreased muscle tone, respiratory complications, and piloerection in mice [50,51]. It was observed that exposure to continuous MPTP resulted in evident behavioral impairments; these impairments were found to be substantially ameliorated by PF pre-treatment. This mitigation was marked by a notable resurgence in TH-positive neuronal cells in the striatum, indicative of PF’s effectiveness in reversing MPTP’s neurotoxic effects on dopaminergic neurons in the substantia nigra striatum. This assertion is further bolstered by protein level assessments. The mechanism underlying PF’s neuroprotection is attributed to its ability to enhance BDNF autocrine signaling, subsequently activating the BDNF/CREB and PI3K/Akt pathways. This activation is crucial for promoting neuronal survival and facilitating tissue repair, thereby establishing PF’s protective role. In summary, our findings provide insight into the potential of TGP in therapeutic implications for PD.

## 4. Materials and Methods

### 4.1. Proteomics Analysis

#### 4.1.1. Total Protein Extraction

PC12 cells were divided into three groups: the Cell group—PC12 cells grown normally without treatment; Model group—MPP^+^ (500 μM); and Drug group—500 μM MPP^+^ + PF-25 (PF-100 μM). At 48 h after the cells were seeded, cultures were refreshed with serum-free medium with the respective drug regimes. After a further 48 h incubation, tests were performed.

In each of the three groups, the sample underwent sonication in a lysis buffer on ice three times, utilizing a high-intensity ultrasonic processor. Subsequently, centrifugation was performed at 12,000× *g* and 4 °C for 10 min to eliminate any residual debris. The supernatant was then collected, and its protein concentration was quantified using a BCA kit (Thermo Fisher Scientific, Waltham, MA, USA), adhering to the guidelines provided by the manufacturer.

#### 4.1.2. Trypsin Digestion

Initially, the protein sample underwent a reduction using 5 mM dithiothreitol at 56 °C for 30 min, which was subsequently followed by alkylation for 15 min at room temperature in darkness, employing 11 mM iodoacetamide. To reduce the urea concentration to under 2 M, the sample was diluted with 100 mM TEAB. The process of proteolytic digestion commenced with the addition of trypsin in a 1:50 trypsin-to-protein ratio overnight, followed by a secondary digestion period lasting 4 h, utilizing a trypsin-to-protein ratio of 1:100.

#### 4.1.3. TMT Labeled Quantitative Proteomics

The trypsin-digested peptides were desalted using Strata X C18 columns (Phenomenex, Torrance, CA, USA) followed by lyophilization. Subsequently, the peptides were reconstituted in 0.5 M triethylammonium bicarbonate (TEAB) buffer and labeled using a TMT 11-plex kit (Thermo Fisher Scientific, Waltham, MA, USA) with three biological replicates per group, assigned as follows: Cell group (126, 127N, 127C), Model group (128N, 128C, 129N), and Drug group (130N, 130C, 131). The labeling reagents were thawed, dissolved in anhydrous acetonitrile, and combined with the peptide solutions. The reaction proceeded at 25 °C for 2 h, and then samples were pooled, desalted, lyophilized for subsequent analysis.

#### 4.1.4. Protein Identification and Quantification

Then the labeled samples were separated by high-performance liquid chromatography in an Agilent 300Extend (Agilent, Santa Clara, CA, USA) C18 column (5 μm particles, 4.6 mm ID, 250 mm length). Mobile phase A was an aqueous solution containing 0.1% formic acid and 2% acetonitrile; mobile phase B was an aqueous solution containing 0.1% formic acid and 98% acetonitrile. The liquid phase gradient was set as follows: 0–26 min, 6–23% B; 26–34 min, 23–35% B; 34–37 min, 35–80% B, and 37–40 min, 80–80% B. The flow rate was maintained at 450 nL/min. The peptides were separated using an UHPLC system, injected into an NSI ion source for ionization and then injected into an Orbitrap Exploris TM 480 mass spectrometer (ThermoFisher Scientific, Waltham, MA, USA) for analysis.

Protein identification and quantification were performed using MaxQuant (v1.5.2.8) with the following parameters: MS/MS spectra were searched against the Rattus norvegicus UniProt reference proteome (database version: 20210721, 29,934 sequences) using Proteome Discoverer (v2.4.1.15). To ensure data quality, reverse decoy sequences were included for false discovery rate (FDR, 1%) calculation, and common contaminant proteins were added to the search database for filtration. The accuracy FDR for identification at the spectral, peptide, and protein levels was set at 1%; identified proteins needed to contain at least one specific peptide, with average ratio-fold change > 1.3 (upregulation) and <0.77 (downregulation), as well as a *p*-value < 0.05.

#### 4.1.5. Functional Analysis of Proteins and DEPs

Bioinformatics tools were employed to conduct further analyses on proteins exhibiting significant differential abundances as observed in the proteomic analysis. A Gene Ontology (GO) annotation proteome was obtained from the Geneontology database (http://www.geneontology.org/; accessed on 26 March 2025). To perform family and pathway studies of functional proteins on the identified proteins, GO and KEGG were utilized. Proteins were classified by GO annotation based on three categories: biological process, cellular component, and molecular function. For each category, the enrichment of DEPs against all identified proteins was tested using a two-tailed Fisher’s exact test. The Kyoto Encyclopedia of Genes and Genomes (KEGG) database was used to identify enriched pathways by a two-tailed Fisher’s exact test to test the enrichment of the DEPs against all identified proteins. The pathways with a corrected *p* value < 0.05 were deemed significant.

### 4.2. In Vitro and In Vivo Experiments

#### 4.2.1. Cell Culture and Treatment

Culture conditions for PC12 cells involved DMEM enriched with a 10% concentration of fetal bovine serum along with antibiotics, specifically 100 units/mL each of penicillin and streptomycin. To promote ideal proliferation, the environment for incubation was regulated to maintain a temperature of 37 °C and an atmospheric composition of 5% CO_2_ with the remainder being air. For the experimental assays, only cells that were actively proliferating in their logarithmic growth phase were selected to ensure consistency in responses. The study involved treating the PC12 cells with a range of PF concentrations (0 to 300 μM) for 24 h to meticulously assess the cytotoxic potential of PF via the MTT assay. To develop an in vitro Parkinson’s disease model, cells underwent exposure to 500 μM of MPP^+^ over 24 h. Initially, to prepare for MPP^+^ exposure (500 μM), cells received treatments with varying doses of PF (25, 50, and 100 μM) or Madopar (50 μg/mL) as a reference control, 48 h in advance. This preliminary treatment aimed to assess the protective capacity of PF and Madopar against the induction of Parkinson’s disease-like effects in PC12 cells, thereby laying the groundwork for further in-depth experimental analysis.

#### 4.2.2. Cell Viability

An MTT assay was used to determine cell viability. After treatment with the drug, cells were incubated with MTT reagent for around 4 h. Then, 100 μL of DMSO was added for solubilizing the formazan crystals. The optical density at a wavelength of 490 nm was determined by means of a spectrophotometer, which was the starting point for the determination of the relative viability of cells.

#### 4.2.3. Lactate Dehydrogenase (LDH) Release Rate Assay

LDH activity in cell culture supernatants was detected by a colorimetric assay. After removing the cell culture fluid from each group, preformulated LDH working solution (LDH Cytotoxicity Assay Kit, Beyotime, Shanghai, China) was added and incubated for 40 min at room temperature away from light. The absorbance value of each well was measured at 490 nm using a multifunctional enzyme marker (BioTek Synergy H1, Agilent, Santa Clara, CA, USA), and the LDH release rate (%) was calculated from the standard curve.

#### 4.2.4. Determination of Intracellular Calcium Ion (Ca^2+^) Concentration

The fluorescent probe Fluo-3/AM (Thermo Fisher Scientific) was applied for intracellular Ca^2+^ dynamic monitoring. After removing the culture medium, 5 μM Fluo-3/AM probe (containing 0.02% Pluronic F-127) was loaded and incubated for 30 min at 37 °C away from light. A fluorescence spectrophotometer (Hitachi F-7000, Hitachi Limited, Tokyo, Japan) was used to detect the fluorescence intensity at an excitation wavelength of 488 nm and an emission wavelength of 525 nm, and the intracellular Ca^2+^ concentration (nM) was quantified by the standard curve for calcium ions.

#### 4.2.5. Reactive Oxygen Species (ROS) Level Detection

Intracellular ROS levels were assessed based on the DCFH-DA fluorescent probe assay (ROS Assay Kit, Beyotime, Shanghai, China). After removing the culture medium, 10 μM DCFH-DA working solution was added and incubated at 37 °C for 40 min away from light, and PBS was washed 3 times to remove residual probe. The fluorescence intensity was measured using a multifunctional enzyme marker (excitation 488 nm/emission 525 nm), and the ROS level was expressed in relative fluorescence units (RFUs).

#### 4.2.6. Apoptosis Rate Detection

Apoptosis was detected by the Annexin V-FITC/PI double-staining method (Apoptosis Detection Kit, BD Biosciences, Franklin Lakes, NJ, USA). Cells were collected from each group, the cell concentration was adjusted to 1 × 10^6^ cells/mL, and 100 μL of cell suspension was taken and incubated with 5 μL of Annexin V-FITC and 5 μL of PI working solution for 15 min away from light. Detection was performed by flow cytometry (BD FACSCalibur, Franklin Lakes, NJ, USA), and the data were analyzed by FlowJo (V10.8.1) software to calculate the proportions (%) of early apoptotic (Annexin V+/PI-) and late apoptotic (Annexin V+/PI+) cells.

### 4.3. Animals

#### 4.3.1. Statement of Ethics

In conducting this research, meticulous adherence to the NIH guidelines for the Care and Use of Laboratory Animals was ensured. Concurrently, ethical endorsement for the study was secured from the IACUC at Changchun Normal University, China. This endorsement was crucial for ensuring the study’s commitment to ethical standards, notably in reducing the number of animals utilized and alleviating any distress they might encounter during the research process.

#### 4.3.2. Animal Behavioral Assessment

In this study, adult male C57BL/6 mice were used, under the authorization SCXK(JING) 2005-0013. Sixty mice were divided into six groups of equal number: Group A acted as the control; Group B was exposed to MPTP; Groups C, D, and E were treated with PF at doses of 7.5, 15, and 30 mg/kg (orally administered), respectively, after exposure to MPTP; and Group F received Madopar (100 mg/kg) following MPTP exposure. The administration of paeoniflorin and Madopar was carried out daily via oral gavage, whereas Groups A and B were given 20 mg/kg/day of double-distilled water intraperitoneally, over a span of 15 days. Starting from the eighth day, mice in the experimental groups, excluding Groups A and B, were given eight intraperitoneal injections of MPTP (30 mg/kg.d). Behavioral assessments, including spontaneous activity and roller tests, were conducted one hour after the last MPTP injection.

#### 4.3.3. Spontaneous Motor Activity Test

To assess spontaneous movements, the study employed the ZIL-2 Spontaneous Activity System (Shanghai, China), a state-of-the-art digital tracking device designed for motion analysis. Before initiating the formal assessment, the mice were allowed a brief acclimation period of three minutes for unrestricted movement, facilitating their adjustment to the testing environment. Subsequent to this initial adaptation phase, the system was then tasked with the precise documentation of the mice’s spontaneous movements, both horizontally and vertically, throughout a meticulously observed five-minute interval [19,20].

#### 4.3.4. Pole Test

In the pole test, a vertical pole (length: 50 cm; diameter: 1 cm) was employed, with a metal ball (diameter: 2.5 cm) affixed to its top. To prevent slippage, the pole was wrapped with layers of gauze. Mice were positioned on the top of the pole with their tail tips pinched and their heads oriented downward. Their hind limbs were carefully positioned to rest on the surface of the ball, after which they were required to climb down the pole in a downward direction. Following a 4-day acclimatization period, the time taken for the mice to descend from the top of the pole to the point where their forelimbs touched the bottom was measured and recorded.

In the conducted experiments, researchers placed mice on a rotating rod and monitored the time elapsed from the commencement of rotation until the mice voluntarily exited the apparatus. Each experimental session was strictly limited to a duration of 180 s, with the daily mean derived from the aggregation of three distinct evaluations.

#### 4.3.5. Immunohistochemistry

For the immunohistochemical analysis, four mice were perfusion-fixed with 4% paraformaldehyde following a heparinized saline flush, 7 days after behavioral assessment. The brains were dissected and post-fixed in paraformaldehyde overnight at 4 °C and then transferred to 30% sucrose in 0.1 M phosphate buffer (PB) at 4 °C for 24 h. A cryostat was used to obtain a series of 20 μm thick coronal sections through the ventral mesencephalon. The nigral brain sections were rinsed in PBS + Triton X-100 (Beyotime, Shanghai, China) (PBST), treated with 3% hydrogen peroxide to quench endogenous peroxidase activity, and incubated in a blocking solution. The sections were then incubated with anti-tyrosine hydroxylase (TH, monoclonal mouse, Abcam, Shanghai, China, 1:200) at 4 °C overnight. Following this, the sections were treated with a biotinylated secondary antibody for 1 h at 37 °C, followed by incubation with streptavidin-peroxidase for 1 h. Subsequently, the sections were stained with 3,4-diaminobenzidine (DAB). The results were analyzed by counting the number of positive cells at 400× magnification using a Nikon microscope (Eclipse Ci-L, Nikon Corp., Tokyo, Japan). The region of interest was captured with a camera and analyzed using Image-Pro Plus 6.0 software. The average number of positive cells was used to represent cell density.

### 4.4. Western Blot Analysis

The cell lysate was prepared with a protease inhibitor concentration of 1 mM PMSF at a ratio of 1:99 to the lysate. Cells were lysed on ice for approximately 20 min before being centrifuged at 4 °C and 13,000× *g* for 5 min. The supernatant was then collected for further sample processing. Following electrophoresis and membrane transfer, the membranes were incubated overnight with primary antibodies targeting p-Akt, t-Akt, t-PI3K, p-PI3K, BDNF, p-CREB, t-CREB, and β-actin proteins at a dilution of 1:1000 at 4 °C. After washing, the blots were treated for 45 min with peroxidase-linked secondary antibodies. Protein levels were detected using the enhanced chemiluminescence (ECL) system. The Quantity One (V4.6.8) software from Bio-Rad Laboratories in Hercules was employed for the analysis, quantification, and normalization of the band intensities relative to β-actin staining.

### 4.5. Statistics

Data presentation was uniform, showcasing averages ± standard deviations derived from a minimum of three distinct experiments. For the comparative analysis of two sets, the Student’s *t*-test or a one-way ANOVA was employed. Meanwhile, to differentiate between multiple groups, Dunnett’s post hoc test was utilized, facilitated by Graph Pad Software (V10.3.0). A *p*-value of less than 0.05 was considered indicative of statistical significance.

## 5. Conclusions

In conclusion, PF notably enhanced the viability of PC12 cells and mitigated the MPP^+^-induced mitochondrial dysfunction, oxidative stress, and apoptosis. Analysis of proteomics suggests that these effects may be mediated through activation of the BDNF/PI3K/AKT/CREB signaling pathway. Both in vitro and in vivo studies corroborate that PF offers protection against Parkinson’s disease models induced by MPP^+^ and MPTP, through the stimulation of the PI3K/AKT and BDNF/CREB pathways. The application of LY294002, an inhibitor of the PI3K/AKT pathway, was observed to curb the neuroprotective efficacy of PF against neuronal damage. This work provides a foundational basis for further exploration into the pathogenesis of Parkinson’s disease and offers insights for the development of novel therapeutics.

## Figures and Tables

**Table 1:** Top ten differential proteins.

Protein Accession	Protein Name	Fold Change
P23928	Alpha-crystallin B chain	6.655
A0A0G2JW88	Microtubule-associated protein (MAP4)	6.131
O35147	Bcl2-associated agonist of cell death (BAD)	4.941
D3ZTF6	Phosphatidylinositol 3-kinase (PI3K)	3.932
Q3HSE5	RAC-alpha serine/threonine-protein kinase (AKT)	3.557
D3ZQX3	Mitochondrial ribosomal protein S12	3.111
Q56A19	Brain-derived neurotrophic factor (BDNF)	2.498
O35532	Methylsterol monooxygenase 1	2.295
G3V9C8	ATP-binding cassette subfamily B member 1A	2.289
A0A0G2JXS3	Nucleolar and spindle-associated protein 1	2.252

## Data Availability

The data that support the findings of this study are available within the article.
